# Polarized Secretion of Interleukin (IL)-6 and IL-8 by Human Airway Epithelia 16HBE14o- Cells in Response to Cationic Polypeptide Challenge

**DOI:** 10.1371/journal.pone.0012091

**Published:** 2010-08-12

**Authors:** Alison Wai-ming Chow, Jocelyn Feng-ting Liang, Janice Siu-chong Wong, Yan Fu, Nelson Leung-sang Tang, Wing-hung Ko

**Affiliations:** 1 School of Biomedical Sciences, The Chinese University of Hong Kong, Shatin, Hong Kong, Special Administrative Region, People's Republic of China; 2 Department of Chemical Pathology, The Chinese University of Hong Kong, Shatin, Hong Kong, Special Administrative Region, People's Republic of China; 3 KIZ/CUHK Joint Laboratory of Bioresource and Molecular Research of Common Diseases, The Chinese University of Hong Kong, Shatin, Hong Kong, Special Administrative Region, People's Republic of China; Sun Yat-Sen University, China

## Abstract

**Background:**

The airway epithelium participates in asthmatic inflammation in many ways. Target cells of the epithelium can respond to a variety of inflammatory mediators and cytokines. Damage to the surface epithelium occurs following the secretion of eosinophil-derived, highly toxic cationic proteins. Moreover, the surface epithelium itself is responsible for the synthesis and release of cytokines that cause the selective recruitment, retention, and accumulation of various inflammatory cells. To mimic the damage seen during asthmatic inflammation, the bronchial epithelium can be challenged with highly charged cationic polypeptides such as poly-l-arginine.

**Methodology/Principal Findings:**

In this study, human bronchial epithelial cells, 16HBE14o- cells, were “chemically injured” by exposing them to poly-l-arginine as a surrogate of the eosinophil cationic protein. Cytokine antibody array data showed that seven inflammatory mediators were elevated out of the 40 tested, including marked elevation in interleukin (IL)-6 and IL-8 secretion. IL-6 and IL-8 mRNA expression levels were elevated as measured with real-time PCR. Cell culture supernatants from apical and basolateral compartments were collected, and the IL-6 and IL-8 production was quantified with ELISA. IL-6 and IL-8 secretion by 16HBE14o- epithelia into the apical compartment was significantly higher than that from the basolateral compartment. Using specific inhibitors, the production of IL-6 and IL-8 was found to be dependent on p38 MAPK, ERK1/2 MAPK, and NF-κB pathways.

**Conclusions/Significance:**

The results clearly demonstrate that damage to the bronchial epithelia by poly-l-arginine stimulates polarized IL-6 and IL-8 secretion. This apically directed secretion of cytokines may play an important role in orchestrating epithelial cell responses to inflammation.

## Introduction

Human airways are lined by a layer of surface epithelium, which are essential to the integrated function (e.g., effective mucus clearance) of the respiratory tract in health and disease [1], [2]. Asthma is now considered to be an inflammatory disorder of the airways [3]. Eosinophils are believed to play a more important role than other inflammatory cells. There is an association between tissue eosinophilia and the airway hyper-responsiveness of asthma. The release of pro-inflammatory cytokines such as IL-4 and IL-5 that leads to the recruitment and infiltration of eosinophils is thus a hallmark of asthma [4]. The eosinophils degranulate, releasing a range of highly charged molecules (e.g., toxic cationic proteins) that damage the respiratory epithelium and account for many of the histopathologic abnormalities of asthma [5].

The airway epithelium participates in inflammation in many ways. The cells can act as target cells that respond to exposure to a variety of inflammatory mediators and cytokines by altering one or several of their functions, such as mucin secretion or ion transport [6]. Damage to the surface epithelium is due to the secretion of eosinophil-derived, highly toxic cationic proteins, such as major basic protein (MBP) [7]. To mimic the damage seen in asthma inflammation, the bronchial epithelium can be challenged with highly charged cationic polypeptides such as poly-l-arginine, which is similar in structure and function to the biologically active moiety of MBP [8]–[10]. Moreover, the surface epithelium itself is responsible for the synthesis and release of cytokines that cause the selective recruitment, retention, and accumulation of various inflammatory cells [3]. IL-6 and IL-8 are two classic proinflammatory cytokines that play important roles in bronchial epithelial function [11], [12]. Certain inflammatory cytokines alter the fluid and electrolyte transport by the airway epithelium [13], [14]. Therefore, asthma can be considered a disease of the bronchial epithelium, which may contribute to the pathophysiology of airway inflammation [15].

Polarized secretion of proinflammatory cytokines is important for establishing a specific microenvironment for airway inflammation. However, little is known about the polarized secretion of proinflammatory cytokines and its underlying signaling pathway in human bronchial epithelia damaged by cationic proteins. The aims of this study were to 1) investigate the inflammatory cytokine profile of human bronchial epithelia 16HBE14o- cells in response to a challenge with the cationic polypeptide poly-l-arginine; 2) study the polarized secretion of IL-6 and IL-8; 3) characterize the role of MAPK and NF-κB signaling pathways in the regulation of IL-6 and IL-8 secretion; and 4) examine the effect of IL-6 and IL-8 on transepithelial chloride (Cl^−^) secretion.

## Methods

### Cell Culture

All experiments were performed using the immortalized cell line 16HBE14o-, which was derived from bronchial surface epithelial cells [16]. Cells were maintained in Minimum Essential Medium as described previously [17]. For cytokine quantification, cells were seeded onto 24-well Transwell-Clear filter inserts (Costar, Cambridge, MA) with a 0.4-µm pore diameter. For transepithelial resistance (TER) measurement and simultaneous measurements of intracellular calcium concentration ([Ca^2+^]_i_) and short-circuit current (*I_SC_*), cells were seeded onto Transwell-col filter membranes as previously described [18], [19]. Cells reached confluence after 10 days with a resistance greater than 150 Ω·cm^2^. For quantitative real-time PCR (qRT-PCR) and other experiments, cells were grown on 6-well culture plates.

### RNA extraction and Real-time PCR

Total RNA was extracted with TRIzol Reagent (Invitrogen, Carlsbad, CA) according to the manufacturer's instructions as described previously [17]. After treatment with DNase (Turbo DNA-free Kit, Ambion, Austin, TX), RNA was reverse transcribed to cDNA using a High Capacity cDNA Reverse Transcription kit (Applied Biosystems, Foster City, CA). Real-time PCR was performed with LightCycler 480 SYBR Green I Master Mix (Roche, Indianapolis, IN) on an iCycler thermal cycler (Bio-Rad Laboratories, CA). Primer sequences were as follows: IL-6 forward primer: 5′-GCACTGGCAGAAAACAACCT-3′, reverse primer: 5′-TCAAACTCCAAAAGACCAGTGA-3′; IL-8 forward primer: 5′-CCAACACAGAAATTATTGTAAAGC-3′, reverse primer: 5′-TGAATTCTCAGCCCTCTTCAA-3′, GAPDH: forward primer: 5′-TGCACCACCAACTGCTTAGC-3′, reverse primer: 5′-GGCATGGACTGTGGTCATGAG-3′. Relative expression of IL-6 and IL-8 was normalized to GAPDH and determined with the Pfaffl method [20]. A standard curve of Ct versus concentration was plotted to obtain the efficiency of each PCR reaction. This efficiency (E) can be obtained by the formula 10^–(1/slope)^, with an acceptable range 1.8–2.2. Each PCR run included a no-template control and a sample without reverse transcriptase. All measurements were performed in duplicate.

### Measurement of transepithelial resistance (TER)

Confluent 16HBE14o- epithelia were used to measure TER as described previously [19]. The monolayers were mounted in an Ussing chamber and bathed in normal Krebs-Henseleit (K–H) solution. A transepithelial potential difference (p.d.) of 1 mV was applied periodically, and the resultant change in current was used to calculate the TER using Ohm's law. After an equilibrium period of 30 min, the cultured epithelia were incubated with 10 µM poly-l-arginine for 1–5 hours with untreated epithelia as control. All TER values were determined after background subtraction (contributed by the filter and bath solution) and multiplied by the surface area of the filter.

### Measurement of cytokine secretion by antibody array and enzyme-linked immunosorbent assay (ELISA)

16HBE14o- cells were incubated with 10 µM poly-l-arginine for 3 hours, and cells incubated with fresh non-serum-containing medium were used for time-matched controls. Cell culture supernatants were collected and centrifuged at 3000×*g* for 10 min at 4°C. The supernatants were then aliquotted and stored at −80°C until further use. The cytokine profile of the cell culture supernatants was analyzed with a RayBio® Human Inflammatory Antibody Array III kit according to the manufacturer's instructions. The membrane in this kit can simultaneously detect 40 different inflammation-related factors, including cytokines, chemokines, soluble cytokine receptors, and growth factors [21], [22]. The membranes were detected using chemiluminescence (Amersham Pharmacia Biotech, UK) for 1 min at room temperature. The membranes were then exposed to Fuji Film (Fuji, Japan) for 2 min. The signal intensity of individual spots was quantified with the FluorChem™ 8000 imaging system (Alpha Innotech Corp.) A positive control signal on each membrane was used to normalize the signal intensities of individual spots being compared on different membranes.

Quantification of IL-6, IL-8, TNFα and RANTES secretion was done with enzyme-linked immunosorbent assay (ELISA). 16HBE14o- cells were exposed to different concentrations of poly-l-arginine, with or without pharmacological inhibitors, for various periods of times. Supernatant samples from the apical and basolateral compartments were collected, and IL-6, IL-8, TNFα (eBioscience, San Diego, CA) and RANTES (R&D Systems, Minneapolis, MN) secretion was assessed with commercially available ELISA kits, according to the manufacturer's protocol. All conditions were measured in duplicate.

### Western Blot

Western blotting was performed as described previously [17]. In brief, cells grown in a 6-well plate were lysed in RIPA buffer (1% NP-40, 0.1% SDS, 0.5% deoxycholic acid, 50 mM Tris-HCl (pH 7.4), 150 mM NaCl) supplemented with protease inhibitor cocktail. The protein samples were transferred to a polyvinylidene fluoride (PVDF) membrane (Immobilon-P, Millipore Corporation, Billerica, MA) and immunoblotted with specific primary antibody. All blots were developed by an enhanced chemiluminescence detection system (Amersham Biosciences, Piscataway, NJ). The following primary antibodies were used: anti-phospho-p38 MAPK (Cell Signaling Technology, Beverly, MA; 1∶500), anti-p38 MAPK (Cell Signaling Technology; 1∶500), anti-phospho-p44/42 MAPK (ERK 1/2) (Cell Signaling Technology; 1∶500), anti-p44/42 MAPK (ERK 1/2) (Cell Signaling Technology; 1∶500), anti-β-tubulin (Santa Cruz Biotechnology, Santa Cruz, CA; 1∶500), and anti-GAPDH (Sigma, St. Louis, MO; 1∶5,000).

### NF-κB translocation assay

Cells were grown in black 96-well glass-bottomed plates (Costar) and stimulated with either vehicle control, 1 µM poly-l-arginine, or an inflammatory cocktail (10 ng/ml each tumor necrosis factor-alpha [TNFα], interferon-gamma [INF-γ], and IL-1β) for 30 min. Measurement of translocation of NF-κB from the cytoplasm to the nucleus was performed using a Cellomics NF-κB Activation Kit™ (Thermo Fisher Scientific, Pittsburgh, PA) according to the manufacturer's validated protocol as described by others [23], [24]. The nucleus was immunostained with Hoechst 33342, and NF-κB was detected with an NF-κB primary antibody and Alexa Fluor 488–conjugated goat anti-rabbit IgG secondary antibody. Microscopic images were captured with the MetaFluor Imaging System (v.7.5, Molecular Devices, Downingtown, PA) equipped with a cooled CCD camera (Quantix, Photometrics, Tucson, AZ). Image analysis was performed using NIH ImageJ software (v.3.91, http://rsbweb.nih.gov/ij/) as described by Noursadeghi M. *et al.* [25]. NF-κB nuclear translocation is represented as an increase in the nucleus:cytoplasm ratio.

### Simultaneous measurements of [Ca^2+^]_i_ and I_SC_

Agonist-induced calcium signals and anion secretion were measured simultaneously in polarized epithelium as described [17]–[19]. Positive *I_SC_* currents are displayed as upward deflections of the traces and were defined as those carried by anions moving from the basolateral to apical compartments. The effects of IL-6 and IL-8 on agonist-evoked *I_SC_* changes were tested by pre-incubating the epithelia with the cytokine for 1–3 days prior to addition of the agonist.

### Materials and solutions

The bicarbonate-buffered K-H solution contained (in mM) NaCl, 117; NaHCO_3_, 25; KCl, 4.7; MgSO_4_, 1.2; KH_2_PO_4_, 1.2; CaCl_2_, 2.5; and d-glucose, 11; pH 7.4 when bubbled with 5% CO_2_/95% O_2_. The low Cl^−^ solution (10 mM) was prepared by isosmotically replacing NaCl, KCl, CaCl_2_, and MgCl_2_ with Na-gluconate, K-gluconate, Ca-gluconate, and Mg-sulphate, respectively. The membrane permeant acetoxymethylester (AM) forms of Fura-2 and pluronic F127 were obtained from Molecular Probes (Eugene, OR). Uridine 5′-triphosphate (UTP), forskolin, and poly-l-arginine were obtained from Sigma-Aldrich. The human cytokine antibody array kit was from RayBiotech Inc. (Norcross, GA). IL-6 and IL-8 ELISA kits were obtained from eBioscience (San Diego, CA), and recombinant human IL-6 and IL-8 were from PeproTech Inc. (Rocky Hill, NJ). All other general laboratory reagents were obtained from Sigma-Aldrich, and all cell culture reagents were obtained from Invitrogen.

### Data analysis

Pooled data are presented as means ± standard errors (S.E.), and values of *n* refer to the number of experiments in each group. Experimentally-induced changes (Δ) in the Fura-2 fluorescence ratio and *I_SC_* were quantified by measuring each parameter at the peak of a response and subtracting the equivalent values measured immediately prior to stimulation. Statistical comparisons between original data before normalization were performed using Student's *t*-test and analysis of variance, where appropriate, with a *p*<0.05 considered significant.

## Results

### Effect of poly-l-arginine on TER

**Figure 1:**
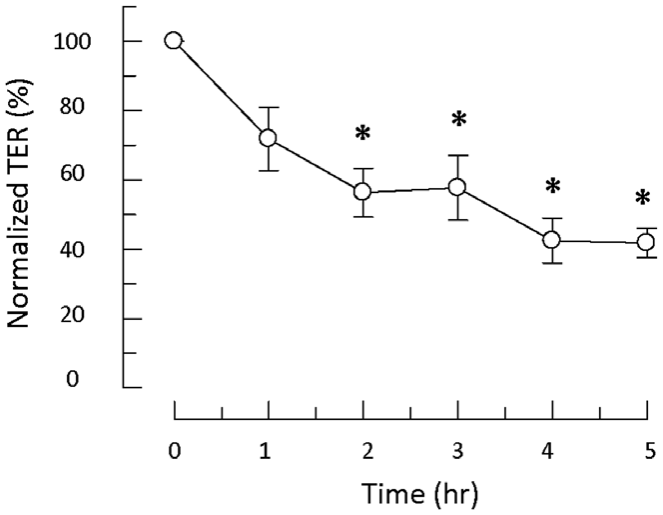
TER measurement during poly-l-arginine exposure. TER was measured at 0, 1, 2, 3, 4, and 5 hours after cells were exposed to poly-l-arginine (50 µM, ap). Each TER measurement was normalized to the TER value of the control epithelia without poly-l-arginine treatment. The data represent the mean ± S.E. (* *p*<0.05, *n* = 4, one-way ANOVA with Scheffe post-hoc test).

TER is an electrical parameter that represents the resistance to ion flow through both paracellular and transcellular pathways. In this study, the effect of poly-l-arginine on TER of cultured 16HBE14o- cells was measured. Fig. 1 shows the time course of the effect of poly-l-arginine on cultured 16HBE14o- cells. The cultured epithelia were incubated with 10 µM poly-l-arginine for 1–5 hours. Each TER measurement was normalized to the initial value without poly-l-arginine treatment. The TER decreased in a time-dependent manner and reached its minimum at 4 hours. Treating the epithelia for 2–5 hours produced a significant reduction in TER. The data therefore confirmed the damaging effect of poly-l-arginine as a surrogate for MBP on TER of the airway epithelium as seen in eosinophilic inflammation [26].

### Poly-l-arginine triggered secretion of cytokines and chemokines by 16HBE14o- cells

**Figure 2:**
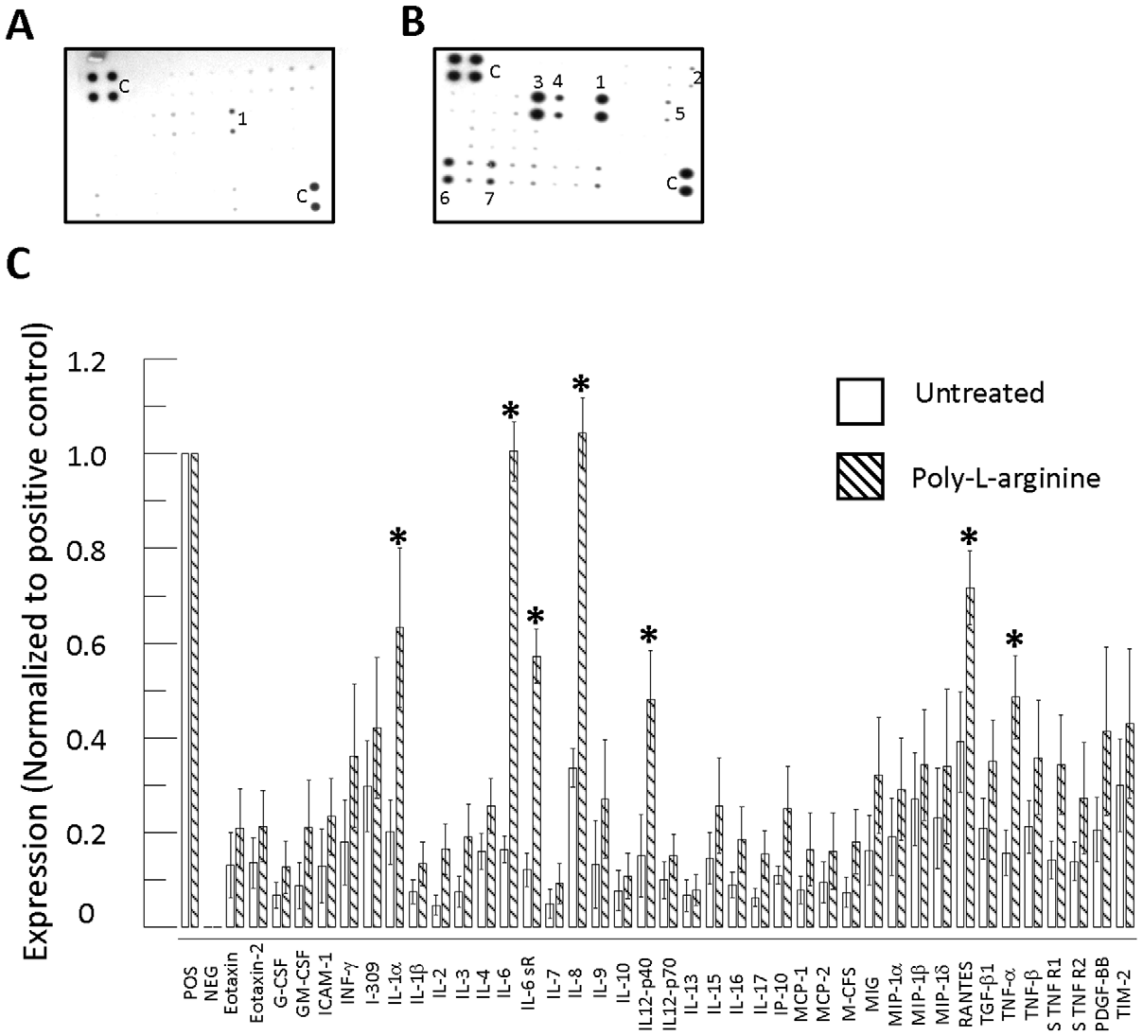
Cytokine and chemokine secretion by 16HBE14o- cells stimulated with poly-l-arginine. 16HBE14o- cells were stimulated with vehicle control (A) or 50 µM poly-l-arginine (B) for 3 hours, and cell supernatants were analyzed using a cytokine antibody array. (C) Summarized data showing the average optical intensity relative to positive control spots. Results represent the mean ± S.E. for 6-7 sets of each antibody array membrane. (c) Internal positive control, (1) IL-8, (2) IL-1α, (3) IL-6, (4) IL-6sR, (5) IL-12p40, (6) RANTES, (7) TNFα. (*, *p*<0.05, Student's *t*-test compared with untreated control).

To further understand the effect of poly-l-arginine in stimulating bronchial epithelial cell cytokine and chemokine secretion, we first examined the cytokine profile in bronchial epithelial cells using the membrane-based RayBio® Human Inflammation Antibody Array III [21], [22]. Fig. 2A and 2B show the control and poly-l-arginine-treated patterns of antibody array images, respectively. The mean optical density of each spot was normalized to the positive control and compared between control and treated epithelia (Fig. 2C). After exposure of the cultured epithelial cells to 10 µM poly-l-arginine for 3 hours, the intensities of two inflammation-related factors (IL-8 and RANTES) increased more than 2-fold, and two other factors (IL-6 and IL-6 soluble receptor [IL-6sR]) increased 5-fold as compared to the control (Fig. 2C). Moreover, the intensities of TNF-α, IL-1α, and IL-12 p40 also increased significantly.

### Apically directed secretion of IL-6, IL-8, TNFα and RANTES by polarized 16HBE14o- epithelia

**Figure 3:**
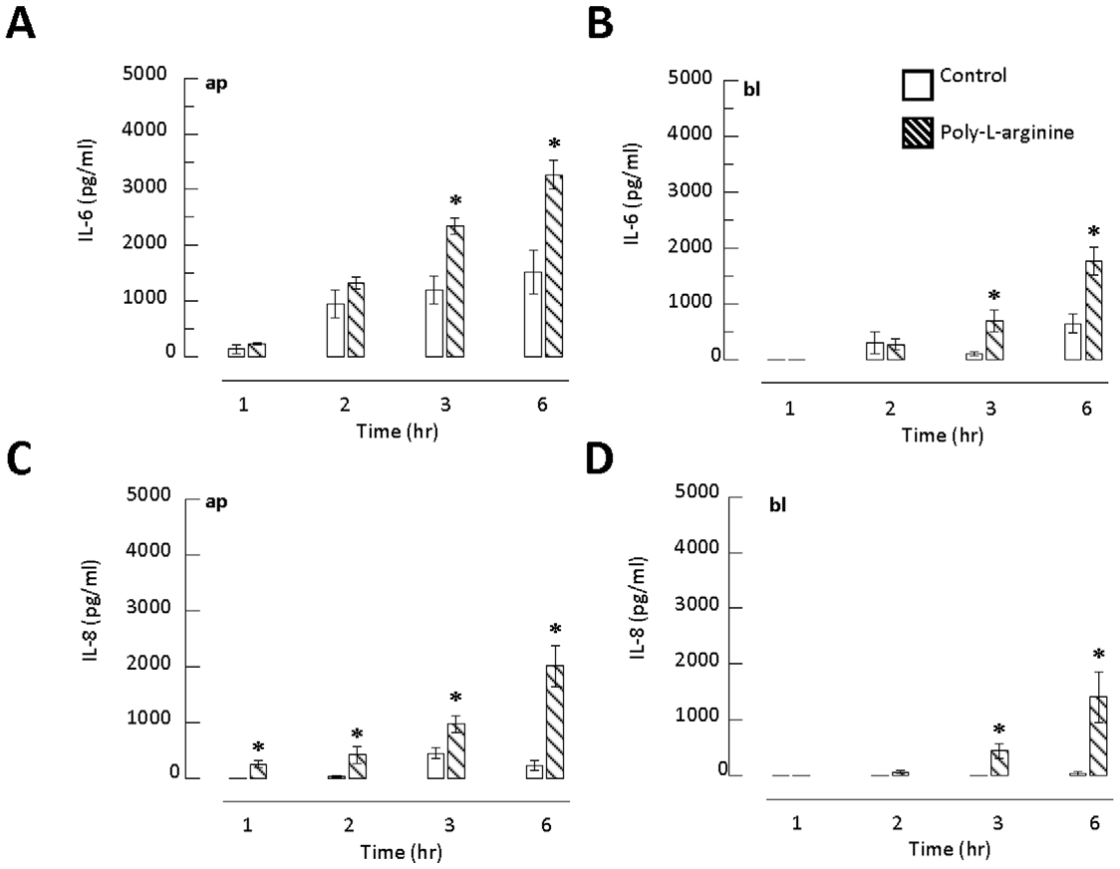
Time course effect of poly-l-arginine on the release of IL-6 and IL-8 into apical (ap) and basolateral (bl) compartments of cultured 16HBE14o- cells. 16HBE14o- cells were exposed to 50 µM poly-l-arginine for various periods of time. Cell culture supernatants in both apical (A and C) and basolateral (B and D) compartments were collected at 1, 2, 3, and 6 hours with time-matched untreated epithelia as control. Each column represents the mean ± S.E. (*n* = 4–9). Statistical significance between the apical or basolateral compartment of the same treatment period is indicated by an asterisk (*p<0.05, Student's *t*-test).

Because epithelia mainly secreted IL-6 and IL-8 into the supernatant in response to poly-l-arginine treatment, we next determined the concentrations of IL-6 and IL-8 secreted by the epithelia. To quantify the polarized secretion of IL-6 and IL-8, supernatant samples obtained from apical or basolateral compartments were analyzed by ELISA. Fig. 3A and 3B show the time course of the effect of poly-l-arginine on IL-6 release into apical and basolateral compartments, respectively. Exposure to 50 µM apical poly-l-arginine stimulated a time-dependent increase in IL-6 release into both apical and basolateral compartments when compared to the time-matched control. Similar results were obtained when we measured the polarized secretion of IL-8 into apical (Fig. 3C) or basolateral (Fig. 3D) compartments.

**Figure 4:**
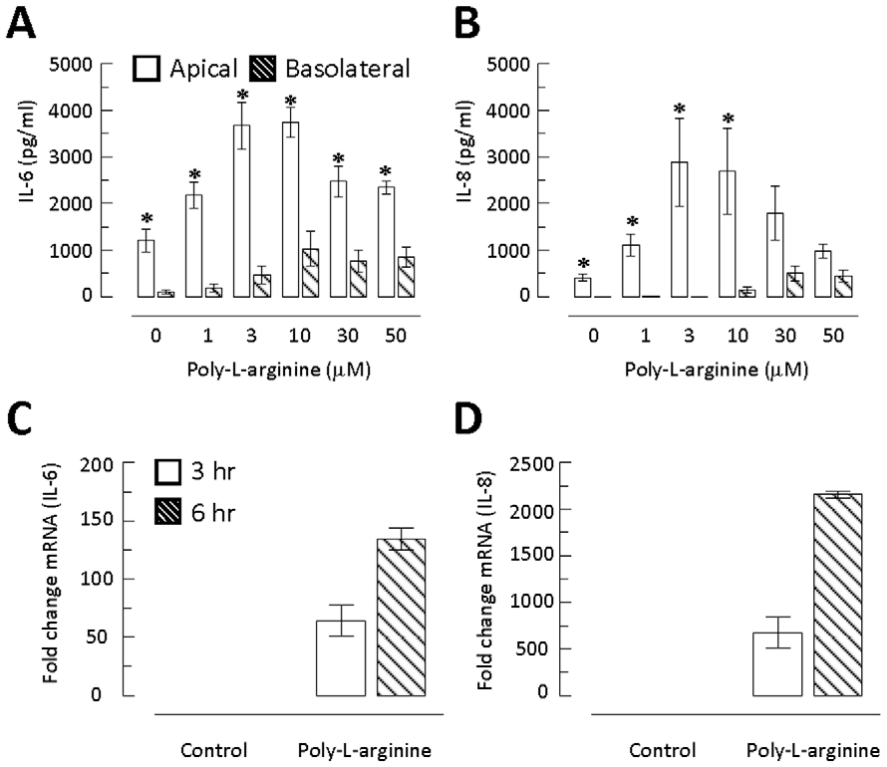
Polarized secretion of IL-6 and IL-8 into the apical compartment. 16HBE14o- cells were exposed to 1–50 µM poly-l-arginine, and cell culture supernatants were collected to measure IL-6 (A) or IL-8 (B) after 3 hours, with untreated epithelia as control. Each column represents the mean ± S.E. (*n* = 3–9). Statistical significance between the apical and basolateral compartments of the same treatment concentration is indicated by an asterisk (* *p*<0.05, Student's *t*-test). mRNA expression of IL-6 (C) and IL-8 (D) was analyzed with qRT-PCR. Epithelia were treated with poly-l-arginine for 3 or 6 hours. Relative expression of IL-6 and IL-8 was normalized to GAPDH and is shown as fold changes relative to untreated controls. Each column represents the mean ± S.E. (*n* = 3).

Fig. 4A shows the concentration effect of poly-l-arginine on IL-6 release in apical and basolateral compartments. Exposure of the cultured epithelia to 1 to 50 µM poly-l-arginine for 3 hours stimulated a concentration-dependent increase from 1 to 10 µM in IL-6 release into both apical and basolateral compartments, which gradually decreased. Exposure to various concentrations of poly-l-arginine for 3 hours induced a significantly higher concentration of IL-6 release into the apical compartment than into the basolateral compartment for all concentrations tested. The data clearly demonstrated polarized secretion of IL-6 into the apical compartment. Similar results were obtained for IL-8 (Fig. 4B), showing that there is a concentration-dependent and predominately apical secretion by the epithelia. To assess if there is significant diffusion of IL-6 or IL-8 from the apical to the basolateral compartment, exogenous recombinant IL-6 or IL-8 (4000 pg/ml) were added to the apical compartment, and their concentrations in the basolateral compartment were measured. After 6 hours, the IL-6 and IL-8 levels were undetectable (*n* = 3), indicating that cytokine secretion into the apical compartment does not significantly contribute to the amount detected on the other side of the compartment.

Experiments were also performed in which poly-l-arginine (50 µM) was added to the basolateral compartment for 3 hours and the IL-6 released into the apical and basolateral compartments was measured. The epithelia secreted a much higher level of IL-6 into the apical (3250±263 pg/ml) than the basolateral (232±46 pg/ml, *n* = 3, *p*<0.05) compartment, suggesting that there is polarized secretion of IL-6, regardless of whether the stimulus was applied from the apical or basolateral side of the membrane.

To demonstrate whether poly-l-arginine treatment affected mRNA expression of IL-6 and IL-8, cells were stimulated for 3 or 6 hours with 10 µM poly-l-arginine, and mRNA expression was measured with qRT-PCR. Increased IL-6 and IL-8 mRNA expression was detected when the cells were stimulated with 10 µM poly-l-arginine (Fig. 4C and 4D). This cationic polypeptide had a more potent effect on IL-8 than IL-6 mRNA expression (>500*-*fold *vs*. 50-fold).

Apart from measuring polarized secretion of IL-6 and IL-8 by 16HBE14o- epithelia, poly-l-arginine – induced TNFα and RANTES secretion into apical and basolateral compartments were also quantified by ELISA. Under basal conditions, there was minimal increase in TNFα secretion in both apical and basolateral compartments while the RANTES level (27.7±6.6 pg/ml) was increased in the apical but not basolateral compartment. When the epithelia were treated with 50 µM poly-l-arginine for 6 hours, there was a significantly higher concentration of TNFα release into the apical compartment (16.1±3.2 pg/ml) than into the basolateral compartment (2.9±1.0 pg/ml, *n* = 3, *p*<0.05). Similarly, the epithelia secreted much higher level of RANTES into apical (101.7±17.9 pg/ml) than the basolateral (24.6±1.3 pg/ml, *n* = 3, *p*<0.05) compartment.

### Polarized secretion of IL-6 and IL-8 is regulated by p38 MAPK-, ERK1/2 MAPK-, and NF-κB-dependent pathways

**Figure 5:**
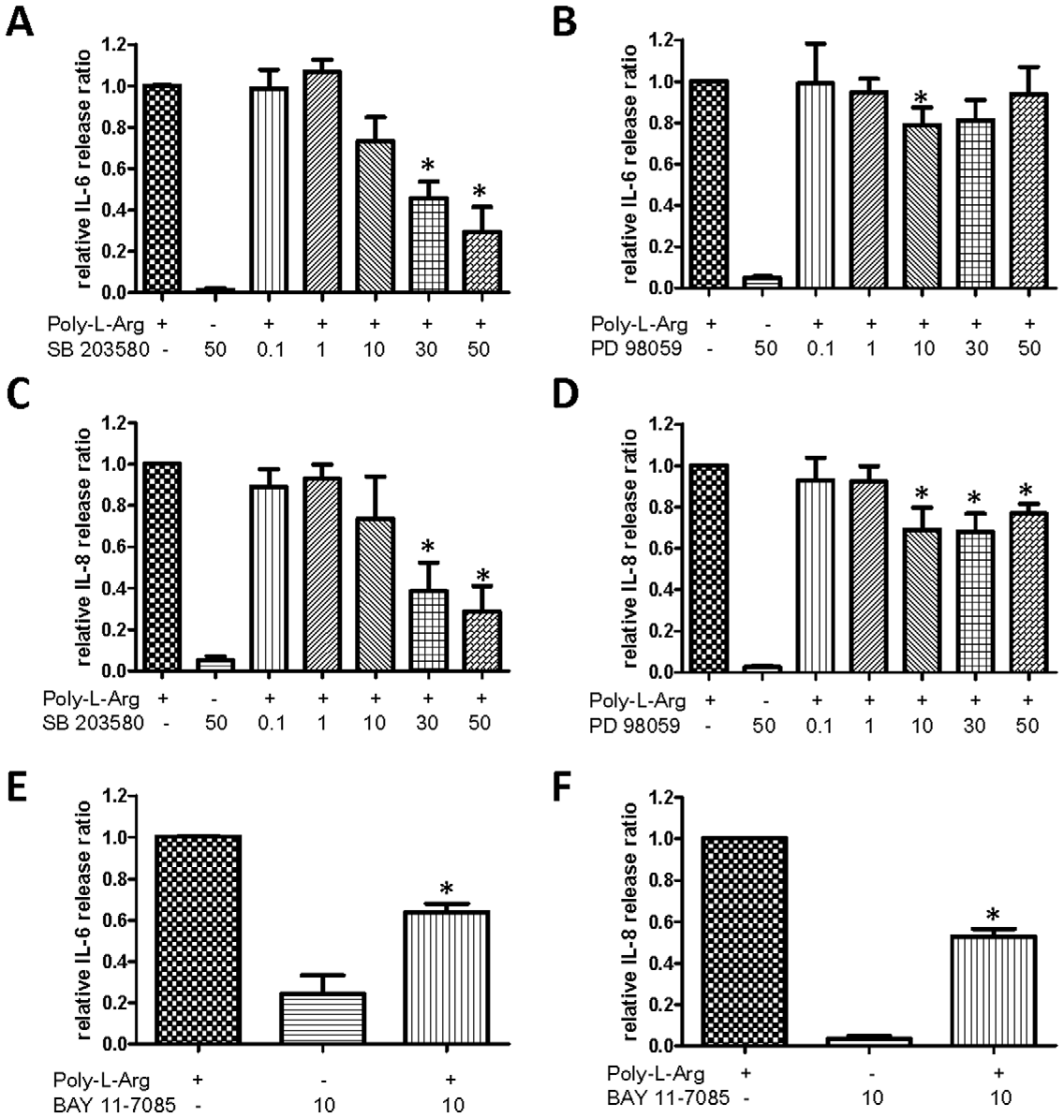
Effect of p38 MAPK, ERK1/2 MAPK, and NF-κB inhibitors on apical IL-6 and IL-8 release by 16HBE14o- cells. Cells were treated with inhibitor alone or co-incubated with poly-l-arginine (10 µM) in the presence of different concentrations of the inhibitors (µM) for 3 hours. IL-6 (A, B, and E) and IL-8 (C, D, and F) levels were measured with ELISA. Levels of IL-6 and IL-8 were corrected with vehicle control alone and normalized to 10 µM poly-l-arginine. Each column represents the mean ± S.E. (*n* = 4). Statistically significant inhibitory effects compared with poly-l-arginine-treated control are marked with an asterisk (*p<0.05, Student's *t*-test).

We next examined the effect of various MAPK and NF-κB inhibitors on poly-l-arginine–induced IL-6 and IL-8 release from 16HBE14o- epithelia because it has been shown that MAPKs (ERK1/2, p38) and NF-κB are involved in IL-6 [27] and IL-8 [28] release from epithelial cells. To evaluate the involvement of these signaling pathways in inducing IL-6 and IL-8 release, selective pharmacological inhibitors were used. For poly-l-arginine–induced apical IL-6 secretion, the p38 MAPK inhibitor, SB203580, suppressed IL-6 production significantly at concentrations of 30 and 50 µM (Fig. 5A). In contrast, the ERK1/2 inhibitor, PD98059, did not show any concentration-dependent inhibition of IL-6 secretion (Fig. 5B), with the exception of an effect at 10 µM PD98059. A similar inhibitory profile was also obtained for apical IL-8 secretion (Fig. 5C and 5D). SB203580 (10 and 50 µM) significantly inhibited IL-8 production, whereas PD98059 (10–50 µM) produced a similar inhibitory effect. The NF-κB inhibitor, BAY 11-7085 (10 µM), also significantly blocked IL-6 (Fig. 5E) and IL-8 (Fig. 5F) production. The effect of these inhibitors on basolateral IL-6 and IL-8 release was erratic, and no clear inhibitory effects were observed (data not shown), possibly due to the small and variable basolateral release of IL-6 and IL-8 compared to the apical release. Taken together, our data suggest that the apical secretion of IL-6 and IL-8 is mediated through p38 MAPK- and NF-κB-dependent pathways. However, the ERK1/2 pathway may also be involved in IL-8 secretion.

### Poly-l-arginine induces p38 and ERK1/2 phosphorylation and NF-κB translocation

**Figure 6:**
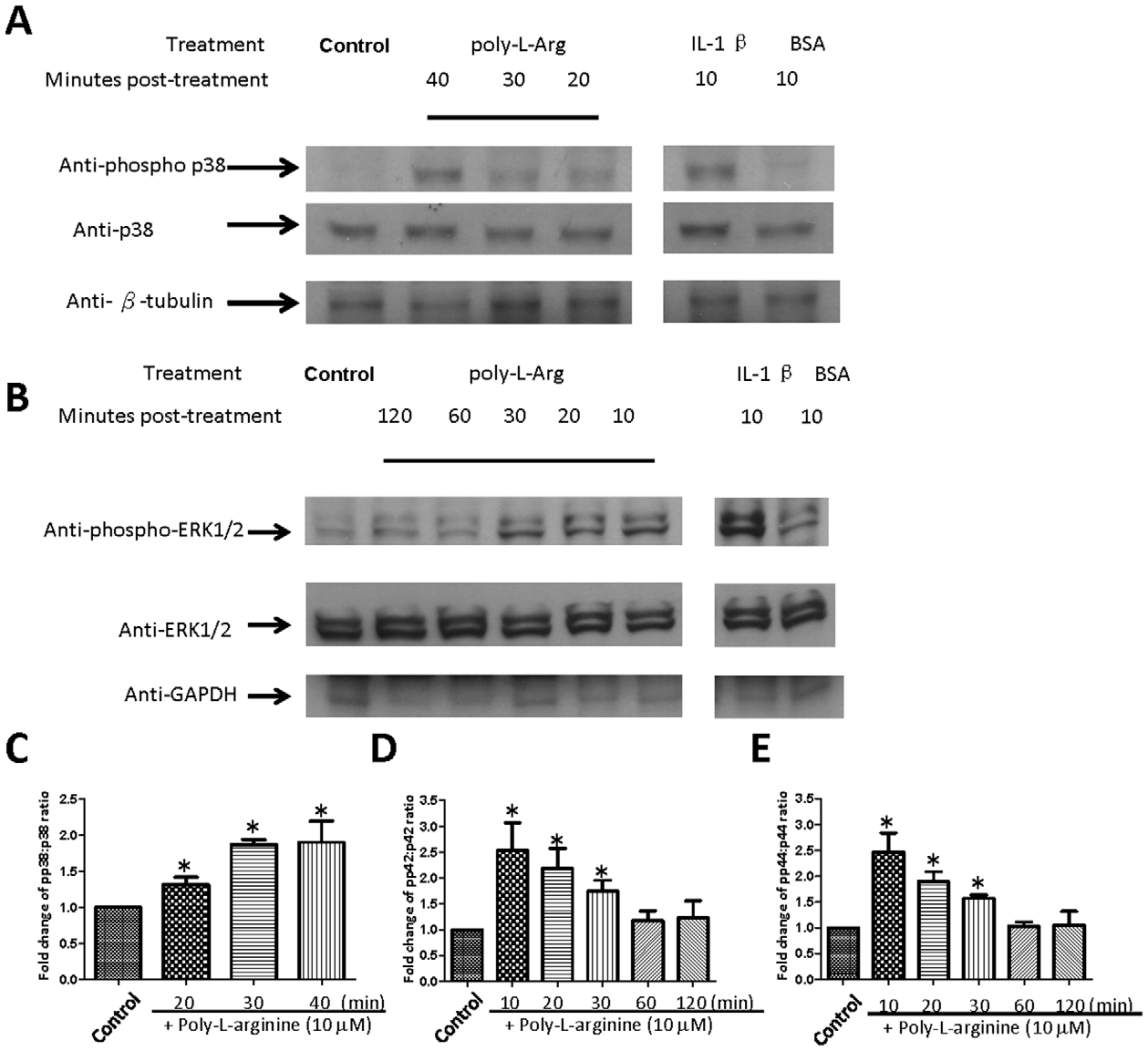
Poly-l-arginine-induced ERK1/2 MAPK and p38 MAPK activation. 16HBE14o- cells were grown on 6-well plates for 9–10 days until confluent and were serum starved for 16 hours prior to treatment. Cells were then treated with 10 µM poly-l-arginine or vehicle control for the indicated periods of time. Total cell lysates were analyzed by SDS-PAGE and immunoblotted with antibodies specific for (A) phospho-p38 MAPK and p38 MAPK or (B) phospho-ERK1/2 MAPK and ERK1/2 MAPK. Quantification of the western blot is shown in (C–E). The band intensity of the phospho-MAPK is divided by that of the total MAPK. The phosphoMAPK:total MAPK ratio of the vehicle control was arbitrarily set to 1. IL-1 β (10 ng/ml) was employed as a positive control, and 0.1% BSA was the vehicle control. Data shown are representative of three independent experiments. (*p<0.05 compared with vehicle control, Student's *t*-test).

To verify that p38 and ERK1/2 MAPK are implicated in IL-6 and IL-8 release when the cells are challenged with poly-l-arginine, the ratio of phosphorylated to total p38 and ERK1/2 MAPK was measured. Poly-l-arginine activated p38 MAPK in a time-dependent manner, with a maximum activation after stimulation for 30 min (Figs. 6A and 6C). Activation of the ERK1/2 pathway was also confirmed by western blotting, which showed that phosphorylated ERK1/2 MAPK was elevated in poly-l-arginine-treated cells (Fig. 6B). Both phosphorylated p42 and p44 were increased after treating the cells with poly-l-arginine for 10–30 min (Fig. 6D and 6E). IL-1β was used as a positive control because it activates p38 [29] and ERK1/2 [30] in 16HBE14o- cells (Fig. 6A and 6B).

**Figure 7:**
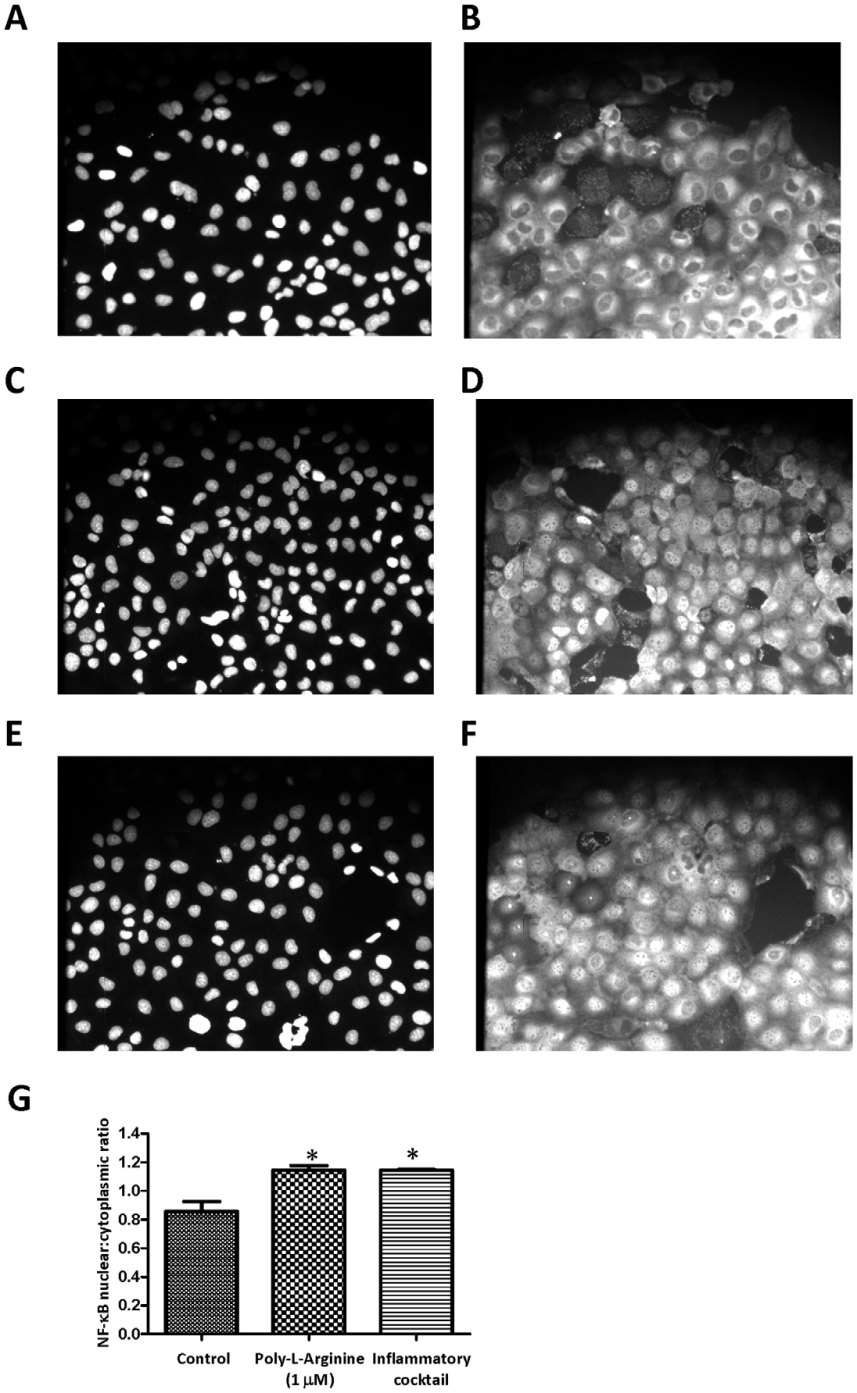
Quantification of poly-l-arginine-induced NF-κB translocation by immunofluorescence staining. 16HBE14o- cells were stimulated with vehicle control (A and B), 1 µM poly-l-arginine (C and D), or an inflammatory cocktail (TNFα, INF-γ, and IL-1β, 10 ng/ml each; E and F) for 30 min. The nuclei (A, C, and E) were stained with Hoechst 33342, and NF-κB (B, D, and F) was stained with anti-NF-κB and Alexa Fluor 488 goat anti-rabbit secondary antibody. (magnification ×20) (G) Immunofluorescence of NF-κB in the nuclear region and cytoplasmic region was quantified using NIH ImageJ software. Nucleus:cytoplasm ratios of NF-κB staining were calculated. Each column represents the mean ± S.E. (*n* = 3). (*p<0.05 compared with vehicle control, Student's *t*-test).

Immunofluorescent staining demonstrated translocation of NF-κB from the cytoplasm to the nucleus and confirmed NF-κB activation upon treatment of the epithelia with poly-l-arginine (Fig. 7A-F). A summary of these data is shown in Fig. 7G. When the cells were treated with poly-l-arginine (1 µM) for 30 min, the nucleus:cytoplasm ratio increased from 0.86±0.07 to 1.15±0.03, similar to the increase observed in cells (ratio  = 1.15±0.005) treated with an inflammatory cocktail consisting of IL-1β, TNFα, and IFNγ [31]. In the presence of BAY 11-7085 (10 µM), NF-κB translocation was completely abolished (0.82±0.05, *n* = 3).

### IL-6 and IL-8 do not affect Ca^2+^- or 3′-5′-cyclic adenosine monophosphate (cAMP)-dependent Cl^−^ secretion in 16HBE14o- epithelia

Our previous study demonstrated that Cl^−^ secretion across the epithelia is mediated through both Ca^2+^- and cAMP-dependent pathways in 16HBE14o- cells [17]. Because certain cytokines may alter Ca^2+^- or cAMP-dependent Cl^−^ secretion [13], [14], the effect of IL-6 and IL-8 on transepithelial Cl^−^ secretion and Ca^2+^ signaling in 16HBE14o- epithelia was monitored using a simultaneous measurement technique.

**Figure 8:**
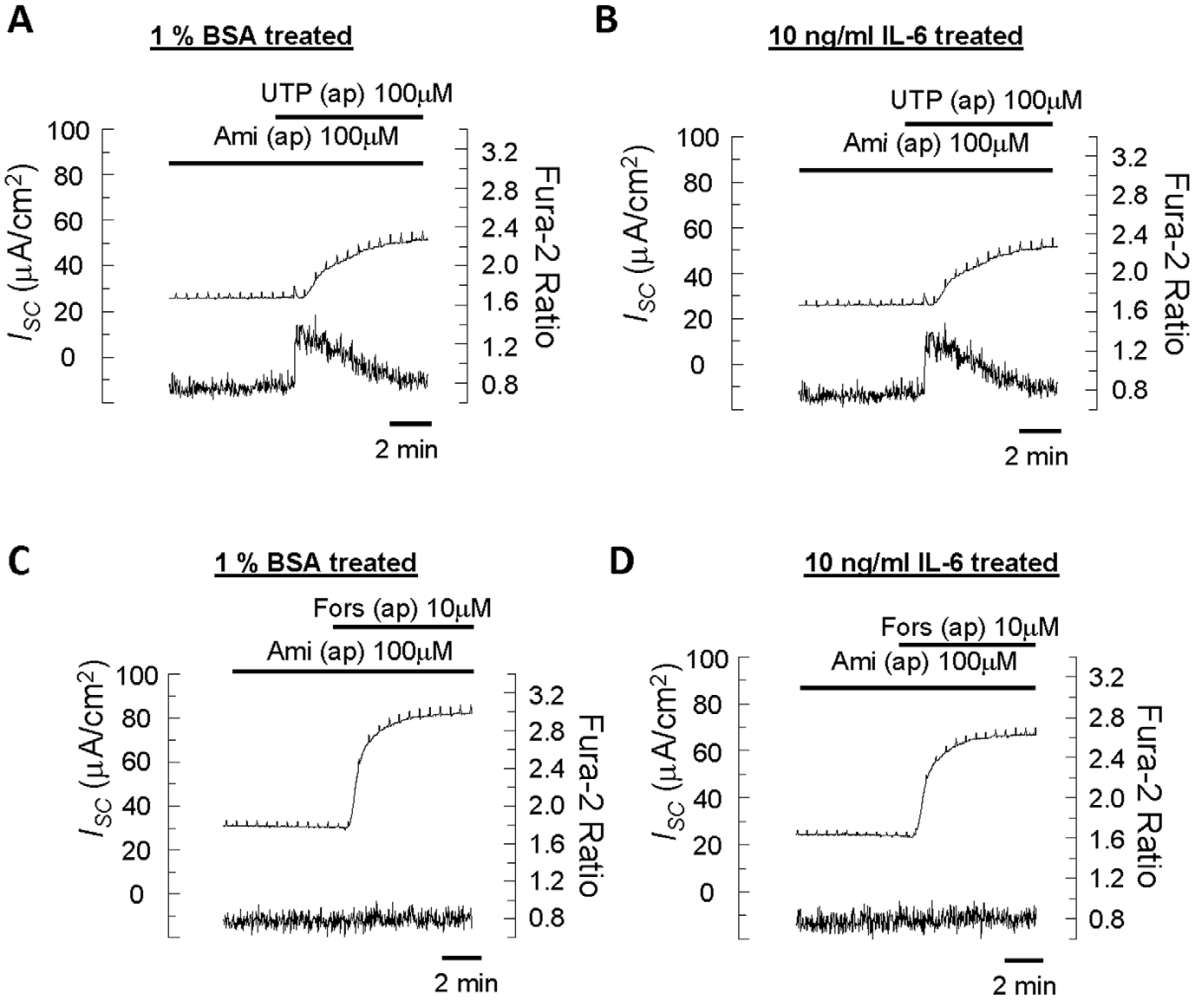
Simultaneous measurement of *I_SC_* and [Ca^2+^]_i_ in 16HBE14o- epithelia treated with IL-6. Representative recordings of simultaneous measurements of *I_SC_* (upper trace) and [Ca^2+^]_i_ (lower trace) in response to apical (ap) application of UTP (A and B) or forskolin (Fors; C and D) in epithelia treated with 1% BSA (A and C) or 10 ng/ml IL-6 (B and D) for 24 hours. 16HBE14o- cells were initially perfused bilaterally with normal K-H solution. The apical K-H solution was then changed to a K-H solution with reduced Cl^−^ concentration (not shown). Cells were then treated with apical application of 100 µM amiloride (Ami) 10 min prior to the apical application of 100 µM UTP or 10 µM forskolin (*n* = 6–8).

Cells were first incubated with vehicle control (1% bovine serum albumin, BSA) or 10 ng/ml IL-6 for 24, 48, or 72 hours. The epithelia were then stimulated with apical application of the Ca^2+^-mobilizing agonist, UTP (100 µM), which activates P2Y_4_ receptors expressed in 16HBE14o- cells [17]. Fig. 8A (control) and 8B (IL-6–treated for 24 hours) show representative recordings of simultaneous measurements of *I_SC_* and [Ca^2+^]*_i_* in response to UTP. Apical application of UTP elicited an increase in *I_SC_*, with a concomitant increase in [Ca^2+^]_i_. Incubation with 10 ng/ml IL-6 did not significantly affect the time course of the maximal *I_SC_* increase (control: 32.1±2.6; treated: 33.6±2.3 µAcm^-2^, *n* = 4–6). Similar results were obtained when epithelia were incubated with IL-6 for 48 (control: 38.3±2.0; treated: 35.0±2.3 µAcm^−2^, *n* = 6, n.s.) or 72 hours (control: 25.8±5.2; treated: 25.1±4.2 µAcm^−2^, *n* = 6, n.s.). Interestingly, incubation with 10 ng/ml IL-6 caused a small, albeit significant decrease in the UTP-induced Fura-2 ratio when the cells were treated with IL-6 for 24 (control: 0.40±0.03; treated: 0.31±0.03, *n* = 4–6, *p*<0.05) or 48 hours (control: 0.41±0.02; treated: 0.28±0.02 (*n* = 6, *p*<0.05), but not when cells were incubated for 72 hours (control: 0.44±0.03; treated: 0.33±0.06, *n* = 6, n.s.).

In another series of experiments, a similar approach was used to examine the effect of IL-6 on cAMP-dependent Cl^−^ secretion. The epithelia were stimulated with the adenylate cyclase activator, forskolin (10 µM), to elevate the intracellular cAMP level. Fig. 8C and 8D show representative recordings of simultaneous measurements of *I_SC_* and [Ca^2+^]_i_ in response to the apical application of 10 µM forskolin in control or treated epithelia (10 ng/ml IL-6 for 24 hours), respectively. Apical application of forskolin elicited an increase in *I_SC_* with no increase in [Ca^2+^]_i_. Incubation with 10 ng/ml IL-6 had no significant effect on the maximal *I_SC_* response (control: 49.9±2.7; treated: 44.5±4.3 µAcm^−2^, *n* = 6). Similar results were obtained when the cells were incubated with IL-6 for a longer period of time (48 and 72 hours, data not shown).

Using the same protocol, cells were incubated with IL-8 (10 ng/ml) for 24–72 hours, and then stimulated with either apical application of UTP or forskolin. Essentially similar effects on *I_SC_* and [Ca^2+^]_i_ were observed (data not shown). In general, chronic exposure of epithelia to IL-6 and IL-8 had no apparent effect on Ca^2+^- or cAMP-dependent Cl^−^ secretion across 16HBE14o- epithelia, despite a slight decrease in nucleotide-evoked [Ca^2+^]_i_ increase.

## Discussion

The airway epithelium participates in inflammation in a number of ways. The surface epithelium itself is responsible for the synthesis and release of cytokines that cause the selective recruitment, retention, and accumulation of various inflammatory cells [3]. Our results confirm that exposing bronchial epithelia to poly-l-arginine mimics the damage caused by MBP in airway inflammation. The decrease in TER may be due to an increase in paracellular permeability as seen in rabbit nasal epithelium [32]. Our antibody array data indicate that challenging the bronchial epithelia with poly-l-arginine resulted in increased levels of multiple proinflammatory proteins including IL-6, IL-6sR, IL-8, TNFα, IL-1α, IL-12p40, and Regulated on Activation Normal T Cell Expressed and Secreted (RANTES). In this study, we focused on the polarized secretion of IL-6 and IL-8 because these two cytokines were increased more significantly than the others. More importantly, these two cytokines are implicated in the initiation and perpetuation of local airway inflammatory responses.

To measure the polarized secretion of IL-6 and IL-8 quantitatively, 16HBE14o- monolayers were grown on a permeable support so that we could collect both apical and basolateral media for analysis. The results strongly indicate that there is a time- and concentration-dependent secretion of IL-6 and IL-8 by the epithelia into the apical compartment compared to the basolateral compartment. Polarized secretion of cytokines, including IL-6 and IL-8, has been observed in other epithelia. Preferential apical secretion was shown in lung (Calu-3 and A549) [33], [34], mesothelial [35], [36], and female reproductive tract epithelia [37]. In contrast, other epithelia, such as renal [38], [39] and colonic [40] epithelia show a preferential basolateral secretion, whereas in retinal pigment epithelium, controversial results were obtained [41], [42]. The direction of polarized secretion therefore appears to be cell-type-specific and may be related to the pathophysiology during inflammation and specific innate immune protection in different tissues.

The polarized secretion of IL-6 and IL-8 (i.e., in an apical-to-basal gradient) may create a chemotactic gradient and induce transepithelial neutrophil migration in a manner analogous to the influx of neutrophils into the airway lumen. Our results strongly suggest that there is apically directed IL-6 and IL-8 secretion in polarized 16HBE14o- epithelia, and that the degree of polarized secretion is always greater in the apical compartment, regardless of whether the stimulus is applied from the apical or basolateral side of the membrane. In fact, the majority of eosinophil degranulation occurs in the lumen of the airway [43]. The exact mechanisms involved in polarized secretion are not fully clear. However, our finding may account for the attraction of neutrophils into the airway lumen following airway inflammatory responses. Together with a decrease in TER and hence barrier function of the epithelial layer upon poly-l-arginine exposure, the polarized secretion of potent chemoattractant would greatly facilitate the migration of immune cells to the lumen of the airway. As a consequence, the polarized secretion of IL-8 may lead to neutrophil recruitment and accumulation, as well as enhancement of IL-8 secretion by neutrophils [44], leading to a positive feedback cycle and progression of airway inflammation. In addition to the apical secretion of cytokines, basolaterally secreted cytokines from epithelia may play another role in supporting the inflammatory response by attracting immune cells from the underlying cellular matrix towards the subepithelial space. Moreover, polarized secretion of TNFα and RANTES by the airway epithelia was observed, which may further aggravate the inflammation and recruit more immune cells, respectively.

In airway epithelial cells, the involvement of p38 MAPK activity in the production of IL-6 [45], [46] and IL-8 [28] has been reported previously. Our experiments demonstrate that the p38 MAPK inhibitor, SB203580, inhibited poly-l-arginine-induced IL-6 and IL-8 release. In addition, the involvement of p38 MAPK was confirmed by western blotting. Poly-l-arginine activated p38 MAPK phosphorylation, with a maximal activation after 30 min. In 16HBE14o- cells, IL-8 release may also be regulated by the ERK1/2 pathway [47], [48]. Although the western blot data confirm a marked increase in p42- and p44–ERK phosphorylation following a poly-l-arginine challenge, the selective inhibitor, PD 98059, only produced a modest inhibitory effect on IL-8 secretion. In contrast, most concentrations of the inhibitor tested did not significantly suppress IL-6 release. Therefore, it appears that although IL-8, but not IL-6 release, may be regulated by an ERK1/2-dependent pathway, the p38 MAPK pathway may be more important in regulating polarized secretion of IL-6 and IL-8. Our study therefore provides a mechanistic explanation of how synthetic polycations induce inflammatory cell migration in the lungs of animal models [49].

Aside from the basal release of IL-6 and IL-8, poly-l-arginine-induced cytokine release into the apical compartment was readily detected 1 hour (IL-8) and 3 hours (IL-6) after stimulation. Our data indicate that within 30 min, the p38 MAPK, ERK1/2 MAPK, and NF-κB pathways were all activated. This relatively short period may indicate that the release originates from an intracellular presynthesized protein. In addition, our results indicate that the cationic polypeptide stimulates IL-6 and IL-8 in a transcription–dependent manner. Earlier studies have shown that cationic proteins stimulate IL-8 mRNA expression in eosinophils [50] and neutrophils [44]. Our results demonstrate a profound upregulation in both IL-6 and IL-8 gene transcription after 3 hours of poly-l-arginine stimulation. The effect was more profound when the cells were treated with poly-l-arginine for 6 hours. The increase in mRNA expression may be due to activation of NF-κB, which is considered to be a crucial inflammatory nuclear transcription factor and a central mediator of inflammatory responses [51]. Previous studies have suggested that both IL-6 and IL-8 secretion in airway epithelia may be regulated in an ERK/NF-κB- [48], [52] or p38 MAPK/NF-κB- [28], [46] dependent manner. Immunofluorescence data from the NF-κB activation assay demonstrates translocation from the cytoplasm to the nucleus after 30 min of poly-l-arginine exposure, similar to that observed with an inflammatory cocktail. In addition, blockade of NF-κB activation by its selective inhibitor, BAY 11-7085, inhibited both IL-6 and IL-8 protein release (∼40–50%), suggesting that the transcription factor NF-κB plays a role in regulating the expression of these two proinflammatory genes. However, transcriptional regulation may not be the only mechanism involved in enhancing cytokine release. In neutrophils, in addition to a transcriptional effect, the cationic protein may also regulate post-transcriptional events by prolonging the stability of mRNA [44]. In fact, p38 MAPK has been implicated in regulating both the post-transcriptional and translational mechanisms required for IL-8 protein expression in intestinal epithelia [53]. Our western blot data and the effect of the selective p38 MAPK inhibitor on IL-6 and IL-8 release strongly suggest that p38 MAPK activation was involved in the secretion process. Because 10 µM BAY 11-7085 did not completely abolish IL-6 and IL-8 secretion but did abolish NF-κB translocation, we cannot exclude the possibility that part of the effect of poly-l-arginine is independent of NF-κB activation and may involve other post-transcriptional mechanisms as seen in intestinal epithelial cells via activation of p38 MAPK [53]. Taken together, our data indicate that whereas NF-κB regulates IL-6 and IL-8 mRNA transcription in airway epithelia, whether the p38 MAPK pathway acts downstream of, and perhaps parallel to, this process awaits further investigation.

Recent studies have suggested that certain inflammatory cytokines affect transepithelial ion transport. In 16HBE14o- cells, both IL-9 and IL-13 augment UTP-induced Cl^−^ secretion via an increased expression of hCLCA1, a Ca^2+^-activated Cl^−^ channel [54]. Therefore, certain cytokines change the balance between fluid absorption and secretion to favor the hydration of the airway surface and, consequently, mucus clearance [14]. However, our study does not support a role for IL-6 or IL-8 in modulating ion transport activities in 16HBE14o- epithelia. Incubating the epithelia for up to 72 hours with IL-6 or IL-8 did not affect Ca^2+^- or cAMP-dependent Cl^−^ secretion across the epithelia when stimulated with UTP or forskolin, respectively. Interestingly, a transient decrease in the UTP-mediated [Ca^2+^]_i_ increase was observed in epithelia treated with IL-6 or IL-8 for 24 to 48 hours. It has been shown that in human airway epithelia, hyperinflammatory responses lead to a larger bradykinin-mediated [Ca^2+^]_i_ increase, which is due to the expanded endoplasmic reticulum/Ca^2+^ store [55]. It appears that IL-6 or IL-8 may not be involved in mediating the inflammation-induced Ca^2+^ store expansion in airway epithelia.

In summary, the cationic polypeptide, poly-l-arginine, induces a time- and concentration-dependent polarized IL-6 and IL-8 secretion towards the apical side of bronchial epithelial cells, effects that are mediated via p38-, ERK1/2-, and NF-κB-dependent pathways. This phenomenon may contribute to the pathophysiology of asthmatic inflammation and the development other inflammatory lung diseases. Further studies on the polarized expression of these cytokines by bronchial epithelia and the detailed underlying signaling mechanism(s) may lead to identification of important therapeutic targets of airway inflammation.
